# *Bian Zheng Lun Zhi* as a Complementary and Alternative Treatment for Menstrual Cramps in Women with Dysmenorrhea: A Prospective Clinical Observation

**DOI:** 10.1155/2014/460386

**Published:** 2014-08-14

**Authors:** Pin-Yi Lin, Yueh-Ting Tsai, Jung-Nien Lai, Chia-Hao Yeh, Ruei-Chi Fang

**Affiliations:** ^1^Institute of Traditional Medicine, School of Medicine, National Yang-Ming University, No. 155, Section 2, Linong Road, Taipei City 112, Taiwan; ^2^Department of Obstetrics and Gynecology, Taipei City Hospital, Yangming Branch, Taipei City 111, Taiwan; ^3^Department of Chinese Medicine, Taipei City Hospital, Yangming Branch, Taipei City 111, Taiwan

## Abstract

*Background*. Limited scientific evidence supports the positive effects of traditional Chinese medicine (TCM) for treating dysmenorrhea. Thus, an observation period of 3 months could verify the ancient indication that TCM treatments effectively alleviate menstrual cramps in women with primary dysmenorrhea or endometriosis. *Methods*. A prospective, nonrandomized study (primary dysmenorrhea and endometriosis groups) was conducted in women with dysmenorrhea for more than three consecutive menstrual cycles. All patients received TCM prescriptions based on *bian zheng lun zhi* theory 14 days before menstruation for a period of 12 weeks. Pain intensity was evaluated using a 10-cm visual analogue scale and two validated questionnaires (the Menstrual Distress Questionnaire and the World Health Organization Quality of Life questionnaire). *Results*. Of the initial 70 intent-to-treat participants, the women with dysmenorrhea reported significant alleviation of cramps during menstruation after the 12-week TCM treatment. Mixed model analysis revealed that TCM prescriptions were more effective in alleviating fatigue, hot flashes, dizziness, painful breasts, excitement, and irritability in the primary dysmenorrhea group (*N* = 36) than in the endometriosis group (*N* = 34). *Conclusion*. TCM prescriptions based on syndrome differentiation theory might be a potentially viable choice for treating painful menstruation and premenstrual symptoms after ruling out endometriosis.

## 1. Introduction

Dysmenorrhea is a gynecological medical condition of pain during menstruation, which is one of the leading causes of women missing work and school [1], and can be classified as either primary or secondary based on the absence or presence of an underlying cause [2]. Primary dysmenorrhea is diagnosed when no underlying cause is detected. Endometriosis, defined as the presence of endometrial tissues at sites other than its normal location in the uterus causing pain or infertility, is the most common cause of secondary dysmenorrhea and one of the leading reasons for regular use of analgesics [3, 4]. Although several studies have suggested that medical and surgical treatments are effective in relieving the pain and accompanying premenstrual symptoms, the side effects associated with chronically administering analgesics and hormonal treatments remain problematic [5, 6]. Notably, the use of alternative therapies for symptomatic relief among women with dysmenorrhea has become nearly as frequent as using conventional therapies [7].

*Bian zheng lun zhi* is one of the most fundamental characteristics of traditional Chinese medicine (TCM), which is a unique approach to diagnosing and treating the pathological process of a disease, and this differentiates TCM from western medicine and other complementary and alternative medicines. The* bian zheng* entails gathering clinical symptoms and signs through the four diagnostic methods (inspection, listening and smelling, inquiry, and pulse-feeling and palpation) and then differentiating disease patterns to confirm a specific type of* zheng* (a pathological summary of the body's health condition at a particular stage in the disease process). Following this diagnostic process, a treatment principle is suggested according to the pattern differentiation; a formula is determined based on the treatment principles, and appropriate herbs are prescribed to achieve the requirements of the determined formula. This process,* lun zhi*, is the standard methodology used in clinical treatment by TCM practitioners.

TCM has been an essential part of health care in Taiwan for hundreds of years and is fully reimbursed under the current National Health Insurance (NHI) system [8, 9]. The Bureau of NHI requires TCM practitioners in Taiwan to diagnose and treat patients based on* bian zheng lun zhi *theory. However, clinical evidence regarding the effects of clinically applying* bian zheng lun zhi* in treating menstrual cramps is lacking. Thus, an observation period of 3 months could disclose any unknown side effects and may verify the ancient indication that TCM therapies effectively alleviate menstrual cramps in women with either primary dysmenorrhea or endometriosis with unconfirmed diagnosis, as mentioned in the classical literature of Chinese medicine.

## 2. Materials and Methods

This prospective, open-label, nonrandomized study was performed between June 2011 and May 2012 at the Yangming branch of Taipei City Hospital, Taipei, Taiwan. All study nurses attended a training session prior to the beginning of the study to ensure standardization of all procedures and fulfillment of good clinical practice (GMP) guidelines. This study was approved by the Taipei City Hospital Institutional Review Board (TCHIRB-1000504-E). Women were recruited from the general population through advertisements, and this trial was conducted according to the Declaration of Helsinki.

### 2.1. Participants

At the initial screening visit, the study participants were women with dysmenorrhea, aged 20–50 years, with a 10-cm visual analogue scale (VAS) score of >3, and with persistence of this condition for at least three consecutive menstrual periods. To be eligible, it was necessary for the women that they have not participated in any other medical trial for at least 3 months before enrollment and that they have discontinued their current medications before the initial screening: 2 weeks for any herbal medications, nonsteroidal anti-inflammatory drugs, antiprostaglandins, narcotics, prescribed psychotropic drugs, and hypnotics; and 12 weeks for estrogens or progestational agents. In addition, participants with suspected cancer, those receiving chemotherapy or radiotherapy, or those with evidence of renal or hepatic dysfunction were excluded from the study. After receiving written and verbal instructions about the type, importance, implications, and duration of the study, as well as information regarding alternative therapies, all willing participants provided informed consent.

### 2.2. Design and Procedure

Participant eligibility, according to the aforementioned selection criteria, was assessed during the initial two clinical visits. Baseline data (information on personal demographics, reproductive history, smoking, alcohol consumption, and quality of life (QOL)) were obtained after the initial screening visit. On the second visit, the participants underwent physical examination and were diagnosed with endometriosis when vaginal or rectal examinations, transvaginal ultrasonography, or laparoscopy confirmed the presence of endometrial tissue outside the uterus. Primary dysmenorrhea was defined as cramping pain in the lower abdomen occurring immediately before or during menstruation, in the absence of pelvic pathology. These data were collected to ensure that each woman met the minimum eligibility criteria and to screen out respondents with potential poor compliance. At the end of the second visit, each eligible woman was interviewed by a TCM practitioner who was blinded to the participant's western medical diagnosis. Considering the long history of TCM, several TCM formulae have been documented in ancient TCM books for their superior effectiveness in treating dysmenorrhea. The TCM practitioner in this study, who had over 20 years of TCM experience, had described from his own perspective prior to the study the principles for selecting the appropriate TCM formula based on* bian zheng lun zhi* theory for treating patients with dysmenorrhea in Taiwan (Table 1). The TCM practitioner was free to diagnose, select the appropriate TCM formulae, and individualize the TCM treatments for the participants with dysmenorrhea during the study period. The details regarding the TCM prescriptions provided in each treatment session were recorded prior to menstruation. The treatment comprised up to three sessions over three consecutive menstrual cycles. All of the TCM prescriptions used in this study were herbal extract powders from GMP-certified pharmaceutical companies in Taiwan, to ensure their compliance with the international market standards of quality and uniformity. No animal products, endangered species, or restricted herbal ingredients were used in this study. Moreover, each prescription was tested for* Escherichia coli*,* Salmonella species* (bacteria count), and heavy metals. The participants were allowed to take analgesics when needed; however, they were requested to record the number of tablets taken each month during the treatment period. Finally, the participants were counseled regarding the potential side effects of the TCM treatment. The project was funded by the Committee on Chinese Medicine and Pharmacy, and the pharmaceutical companies supplied the TCM formulae and were not involved in any other sponsorship, study design, or monitoring of the participants.

### 2.3. Efficacy/Tolerability

The primary objective of this study was to compare the changes in pain symptoms after a 12-week TCM treatment period between primary dysmenorrhea and endometriosis groups, which was measured using a 10-cm VAS for pain, the left extreme of the scale indicating the absence of pain and the right extreme indicating the maximal possible pain. The study revealed two secondary outcome parameters: first, the World Health Organization Quality of Life questionnaire, the Taiwan brief version (WHOQOL-BREF); and second, the Menstrual Distress Questionnaire (MDQ) that was developed to gain further insight into each participant's personal physiological changes and the psychological impacts commonly experienced prior to or during menstruation, comprising 46 items rated on a 6-point ordinal scale from 1 (*no symptom*) to 6 (*present*,* severe*). For this questionnaire, higher scores were interpreted as symptoms that were more severe [10]. This tool has an adequate internal reliability, validity, and consistency for clinical and community samples of women suffering from premenstrual symptoms [11]. The Taiwan version of the WHOQOL-BREF comprises four domains (physical, psychological, social, and environmental) containing 26 facets and two national items regarding overall QOL and general health [12]. Each item was scored on a 5-point Likert scale, with a higher score indicating a favorable condition. To standardize the domain scores for comparison, the average score of each domain was calculated and then multiplied by 4. Thus, the domain scores ranged from 4 to 20, with a higher score indicating a higher QOL in the corresponding domain.

### 2.4. Statistical Analysis

All statistical analyses were performed using Statistical Package for the Social Sciences (version 18). We used descriptive statistics to summarize the continuous variables and calculated the means (SD) and percentages for continuous or categorical data. The significance of differences in the baseline demographics and clinical characteristics between the primary dysmenorrhea and the endometriosis groups were determined using either the chi-square test or the independent *t*-test. All subsequent data analyses involved using an intention-to-treat (ITT) approach. The primary efficacy variable was the mean change from the baseline in the VAS for pain at weeks 4, 8, and 12. The types of dysmenorrhea were divided into primary dysmenorrhea and endometriosis. The QOL scores were measured using the WHOQOL-BREF. In addition, the severity of menstrual cramps and premenstrual symptoms was measured using the MDQ. The relationship between the scores and the elapsed time (weeks 4, 8, and 12) was assumed to be linear, and the resulting intercepts and slopes were used in a linear mixed-effects model. The fixed parameters of intercept and slope were assumed to be the same for all of the participants, whereas the random effects were considered subject-specific regression coefficients. The three repeated measurements for each participant were assumed to be pairwise correlated. Data analysis of the repeated measurements permitted comparing the response trends over time regarding the administered treatments. The model estimate of the fixed effects of treatment over months was examined using *t*-statistics, whereas F-statistics was used for determining the equality of the slopes of the two treatment-time relationships. The statistical inferences were two-sided for determining the fixed effects, and *P* < 0.05 was considered statistically significant.

## 3. Results

**Figure 1:**
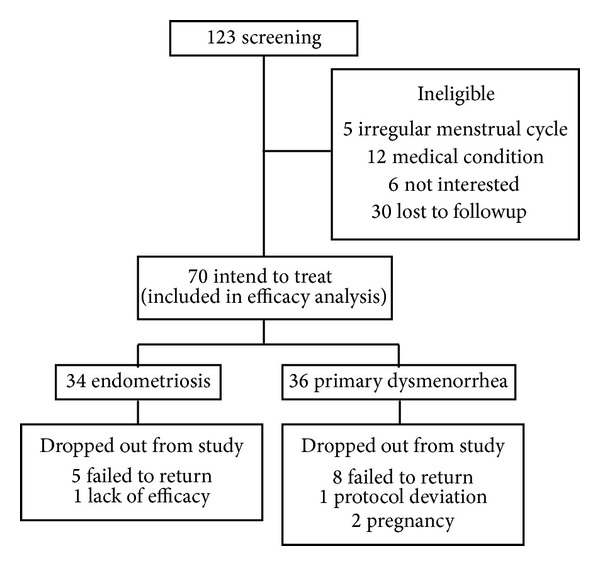
Numbers of participants in study stages.

Of the 123 recruited participants, 53 were ineligible. The primary reasons for ineligibility were as follows: uninterested in participation after a detailed explanation (*n* = 6), irregular menstrual cycles (*n* = 5), medical conditions (*n* = 12), and loss to followup after the initial visit (*n* = 30). Overall, 53 (75.7%) of the initial 70 participants intending to treat completed the 12-week study (endometriosis, *n* = 28; primary dysmenorrhea, *n* = 25). Reasons for withdrawal were as follows: lack of efficacy (*n* = 1) and failure to return (*n* = 5) in the endometriosis group; protocol violation (*n* = 1), pregnancy (*n* = 2), and failure to return (*n* = 8) in the primary dysmenorrhea group (Figure 1).

Table 2 summarizes the demographic and clinical characteristics of the study participants; the mean age, marital status, and analgesic use were significantly higher in the endometriosis group than in the primary dysmenorrhea group. A chi-square test or an independence *t*-test revealed no significant difference in the clinical data between both groups.

After the 12-week TCM treatment period, 20 (58.8%) of the 34 participants in the endometriosis group reported no change in menstrual cramps, 9 (26.5%) reported alleviation, and 3 (8.8%) reported no more menstrual cramps, whereas 2 (5.9%) reported deterioration. Furthermore, 13 (36.1%) of the 36 participants in the primary dysmenorrhea group reported no change in menstrual cramps, 9 (25.0%) reported alleviation, and 10 (27.8%) reported no more menstrual cramps, whereas 4 (11.1%) reported deterioration.

An ITT analysis of the participants in both groups (Table 3) revealed a significant reduction in the VAS pain scores, duration of pain, and menstrual volume; however, no significant difference was observed in the use of analgesics between the baseline and the 12-week follow-up visits. The alleviation of pain severity during menstruation was corroborated by an increase in the global scores of the WHOQOL-BREF. The MDQ scores demonstrated a statistically significant improvement on the pain subscale (cramps, backache, fatigue, and general aches and pains), concentration subscale (low judgment), behavioral change subscale (decreased efficiency), autonomic reaction subscale (dizziness, cold sweats, and nausea), control subscale (chest pains, heart pounding, and fuzzy vision), water retention subscale (painful breasts), and negative affect (anxiety, restlessness, irritability, and mood swings) in both the endometriosis and primary dysmenorrhea groups. However, further analysis revealed that the scores for fatigue, dizziness, hot flashes, painful breasts, irritability, and excitement related to the MDQ were significantly improved in the primary dysmenorrhea group compared with the endometriosis group (Table 4).

## 4. Discussion

Although we used self-comparison to exclude potential confounding by body mass index, smoking, exercise, and socioeconomic status, the possibility of a placebo effect might still exist. Nevertheless, we offer the following arguments supporting the alleviation of painful menstruation through the TCM treatment. First, the participants with dysmenorrhea in both groups reported significant alleviation of cramps during menstruation, and the MDQ and VAS pain scores decreased over time (Table 3). Moreover, improved scores on items of the MDQ and WHOQOL-BREF, including backache, fatigue, general aches and pains, and general health, confirmed the alleviation of abdominal cramps during menstruation (Tables 3 and 4). Although at the end of the study, no significant differences were observed in either group regarding the beneficial effects of the TCM treatment on abdominal cramps, menstrual duration, menstrual flow, and duration of pain, considerable improvement was observed on the control and water retention subscales, and negative affect during menstruation was noted in the primary dysmenorrhea group at the 4-, 8-, and 12-week follow-up points (Table 4). Such effects cannot generally be interpreted as a placebo effect, because the time effect in both groups had already been controlled by the mixed-effects model. Third, the time trend of the reduced severity of the premenstrual symptoms following the TCM treatment may confirm the hypothesis that the Chinese herbs mediated the fluctuations in the participants' hormonal milieu, thus resulting in less frequent accompanying symptoms, lighter menstrual flow, and fewer cramps, particularly in the primary dysmenorrhea group. Thus, we tentatively concluded that the TCM treatment likely alleviates menstrual cramps and that the data merit corroboration in large-scale clinical trials.

Although we cannot exclude a placebo effect, we observed the following positive effects of the TCM treatment on endometriosis: a 35.3% alleviation of pain and a 20.6% decrease in menstrual flow. By contrast, patients with endometriosis did not reduce their frequency of analgesic use, still avoided social activity, and preferred to stay at home during menstruation after the 12-week TCM treatment. Compared with currently available therapies, the TCM treatment may be less effective in reducing the severity of endometriosis-related pain symptoms; however, at the end of the study, the participants reported a 17.8% improvement in work or academic performance. Nevertheless, the long-term administration of analgesics and/or endocrine therapies is of concern, although only 8.8% of the participants with endometriosis perceived a pain-free period following the TCM treatment. This treatment may be appropriate for women who are unable to tolerate the side effects of either medical or surgical treatments for endometriosis.

Although the concepts of* bian zheng*, including TCM theory and methodology, are quintessential in TCM and its concrete applications, the present study revealed that this traditional diagnostic method did not differentiate primary dysmenorrhea from endometriosis when women with dysmenorrhea were treated. Notably, endometriosis is a western medical diagnosis, and a traditional Chinese medical diagnosis is achieved by discerning a characteristic pattern of pelvic pain that has the quality of boring in, is fixed and stabbing in the lower abdomen, and presents dark blood and dark clots in menstrual bleeding, all of which are extremely common symptoms in patients with either endometriosis or primary dysmenorrhea. Based on the study results of applying* lun zhi* (the determination of prescriptions based on syndrome differentiation), the significant differences in positive effects between both groups imply that the diagnostic classification of dysmenorrhea in western medicine might be significant for a TCM practitioner. Moreover, neither group experienced any serious adverse events after treatment cessation based on* lun zhi* theory. Therefore, we propose the following suggestions for improving the quality of health care in treating dysmenorrhea by using TCM. First, the TCM formulae prescribed by TCM practitioners based on the concepts of* bian zheng lun zhi* might be a potential alternative for young women with primary dysmenorrhea who do not experience untoward side effects or have long-term morbidities caused by standard hormonal regimens and analgesics [2]. Second, for women with endometriosis, TCM practitioners should consider the possibility of surrounding organ involvement, which, by extension, may not always correlate with symptom severity, before prescribing herbal formulae to women with dysmenorrhea to mimic the unpredictable effects of TCM treatment. Third, for women with endometriosis who are unable to tolerate the side effects associated with the chronic administration of analgesics and hormonal treatments or those with recurrent endometriosis even after surgical treatment [13], TCM practitioners should proactively observe the efficacy of TCM treatments based on a sequential followup of the serum levels of carbohydrate antigen 125 and ultrasound size measurements.

Following the WHO recommendations, we used the WHOQOL-BREF, a multidimensional measure of QOL, as a secondary outcome in this study. However, no statistically significant changes were observed in the scores of all WHOQOL domains and their various facets for the women suffering from dysmenorrhea who exhibited significant alleviation of their menstrual cramps after the TCM treatment. A major reason for this result is that the mean WHOQOL-BREF scores at the baseline were comparatively higher. Consequently, expecting a further marked increase in such scores may be unrealistic. Another explanation was that, considering its generic nature, the WHOQOL-BREF domain scores may not be sensitive or adequately responsive for detecting such changes.

Notably, a major limitation of our study is its lack of a placebo group and the small study size. Therefore, our claim regarding the efficacy of TCM treatments for dysmenorrhea might not be as reliable as that based on data from a randomized, placebo-controlled clinical trial. However, the present outcome evaluation study avoids the ethical dilemma posed by administering a placebo to symptomatic women who could receive valid treatment and provides valuable evidence for future studies on endometriosis and primary dysmenorrhea that focus on the efficacy of TCM treatments.

In conclusion, TCM treatments based on* bian zheng lun zhi* theory might be a potentially viable choice for treating painful menstruation and premenstrual symptoms, and a differential diagnosis to rule out endometriosis would be helpful. Future studies could evaluate the potential effects of individual TCM formulae in treating painful menstruation and premenstrual symptoms.

## Figures and Tables

**Table 1:** Summary of traditional Chinese medicine diagnoses (*bian zheng* or symptom patterns) and pattern-matching formulas (*lun zhi*) for treating menstrual cramps.

*Bian zheng*	*Lun zhi*		
Symptom and signs	Treatment principle	TCM prescription	Composition
(i) Sharp shooting pain in lower abdomen may precede menstruation by several days and accompany it as menstruation tapers off (ii) Usually coexist with excessively heavy blood loss (iii) Or masses in the abdomen	To activate blood circulation and to remove blood stasis and mass in the abdomen	*Gui-Zhi-Fu-Ling-Wan* (Cinnamon Twig and Poria Pill)	*Cinnamomi Ramulus, Moutan Cortex, Paeoniae Rubra Radix, Persicae Semen, Poria *

(i) Dull pain and distension of the lower abdomen (during menstruation) usually subsides as menstruation tapers off (ii) Usually bleed more frequently (iii) Pain is relieved with warmth but not with pressure	To warm the uterus and expel cold while invigorating and nourishing blood	*Shao-Fu-Zhu-Yu-Tang* (Fennel Seed and Corydalis Combination)	*Angelicae Sinensis Radix, Cinnamomi Cortex, Corydalis Tuber, Foeniculi Fructus, Ligusticum Rhizoma, Myrrha, Paeoniae Rubra Radix, Trogopterori Faeces, Typhae Pollen, Zingiberis Rhizoma *

(i) Stabbing pain of fixed location over the lower abdomen (during menstruation) (ii) Usually coexist with menstrual headache or chest discomfort (iii) Frequently become *agitated* (iv) More likely to be a poor sleeper	To invigorate the blood and expel blood stasis while regulating Qi and unblocking the channels	*Xue-Fu-Zhu-Yu-Tang* (Persica and Carthamus Combination)	*Angelicae Sinensis Radix, Asini Gelatinum, Evodiae Fructus, Cinnamomi Ramulus, Ginseng Radix, Glycyrrhizae Radix, Ligusticum Rhizoma, Moutan Cortex, Ophiopogonis Radix, Paeoniae Alba Radix, Pinellia Tuber, Zingiberis Rhizoma *

(i) Dull pain in lower abdomen may precede menstruation by several days and usually subsides as menstruation tapers off (ii) Usually bleed more frequently (iii) Weak and cold lower part of the body with difficulty in conception (iv) Pain is relieved with warmth or/and with pressure	To warm the uterus and expel cold while invigorating Qi and nourishing blood	*Wen-Jing-Tang* (Tangkuei and Evodia combination)	*Achyranthis Radix, Angelicae Sinensis Radix, Aurantii Fructus, Bupleuri Radix, Carthami Flos, Glycyrrhizae Radix, Ligusticum Rhizoma, Paeoniae Rubra Radix, Persicae Semen, Platycodi Radix, Rehmannia Rhizoma *

(i) Dull pain in lower abdomen may precede menstruation by several days and usually subsides as menstruation tapers off (ii) Usually coexist with menstrual headache or dizziness (iii) lower extremity swelling with difficulty in conception (iv) Pain is relieved with pressure	To nourish the blood and soothe the liver, fortify the spleen, and eliminate dampness	*Dang-Gui-Shao-Yao-San* (Tangkuei and Peony powder)	*Alismatis Rhizoma, Angelicae Sinensis Radix, Atractylodis Ovatae Rhizoma, Ligusticum Rhizoma, Paeoniae Alba Radix, Poria *

(i) Throbbing pain in lower abdomen may precede menstruation by several days and usually subsides as menstruation tapers off (ii) Weak lower part of the body with achy legs	To tonify the Qi	*Shao-Yao-Gan-Cao-Tang* (Peony and Licorice combination)	*Glycyrrhizae Radix, Paeoniae Alba Radix *

(i) Throbbing, nauseating pain may last until the end of menstruation (ii) Usually coexist with excessively heavy blood loss (iii) Dizziness, fatigue, and loss of appetite (iv) Watery discharge expelled from the vagina	To replenish Qi, invigorate the spleen, nourish the heart, and calm the mind	*Qui-Pi-Tang* (Ginseng and Longan combination)	*Angelicae Sinensis Radix, Astragali Radix, Atractylodis Ovatae Rhizoma, Ginseng Radix, Poria, Glycyrrhizae Radix, Longan Arillus, Polygalae Radix, Vladimiria Radix, Zingiberis Rhizoma, Zizyphi Spinosi Semen, Zizyphus Fructus *

(i) Dull pain in lower abdomen and distending pain in the breasts may precede menstruation by several days and usually subsides as menstruation tapers off (ii) Usually coexist with menstrual headache or chest oppression/pain (iii) Frequently become *agitated *	To spread the liver Qi, strengthen the spleen, nourish the blood, and clear the heat	*Jia-Wei-Xiao-Yao-San* (augmented rambling powder)	*Angelicae Sinensis Radix, Atractylodis Ovatae Rhizoma, Bupleuri Radix, Poria, Gardeniae Fructus, Glycyrrhizae Radix, Menthae Herba, Moutan Cortex, Paeoniae Alba Radix, Zingiberis Rhizoma *

(i) Dull pain (during menstruation) in lower abdomen may precede menstruation by several days and usually subsides as menstruation tapers off (ii) Usually coexist with breast swelling and tenderness or shortness of breath (iii) Dizziness, fatigue, and loss of appetite	To regulate the liver Qi, tonify the spleen, and nourish the blood	*Xiao-Yao-San* (Tangkuei and Bupleurum formula)	*Angelicae Sinensis Radix, Atractylodis Ovatae Rhizoma, Bupleuri Radix, Poria, Glycyrrhizae Radix, Menthae Herba, Paeoniae Alba Radix, Zingiberis Rhizoma *

(i) Sharp shooting pain in lower abdomen may precede menstruation by several days and accompany it even menstruation tapers off (ii) Usually coexist with menstrual migraines and breast lumps (iii) Yellowish, foul smelling discharge expelled from the vagina (iv) Swollen and itchy genitalia (v) Difficult and painful urination with a heat sensation in the urethra	To drain damp and clear the heat from the lower burner (the Liver, intestines, bladder, and kidneys)	*Long-Dan-Xie-Gan-Tang* (*Gentiana* drains the liver decoction)	*Akebia Caulis, Alismatis Rhizoma, Angelicae Sinensis Radix, Bupleuri Radix, Gardeniae Fructus, Gentianae Radix, Glycyrrhizae Radix, Plantaginis Semen, Rehmannia Rhizoma, Scutellariae Radix *

**Table 2:** Baseline demographic and clinical characteristics for the 70 participants.

Definition	Endometriosis *N* = 34	Primary dysmenorrhea *N* = 36	*P* value
Age (years)	35.7 ± 4.7	29.5 ± 6.2	0.000∗
Body mass index (kg/m^2^)	20.5 ± 2.4	19.5 ± 2.2	NS
Marital states (%)			
Unmarried	13 (38.2)	24 (66.7)	0.033∗
Married	19 (55.9)	12 (33.3)	
Divorced/widowed/separated	2 (5.9)	0 (0.0)	
Education level (%)			NS
Junior high or below	7 (20.6)	4 (11.1)	
University or above	27 (79.4)	32 (88.9)	
Menstrual duration	2.9 ± 0.8	2.7 ± 0.6	NS
Blood volume	36.4 ± 38.8	26.1 ± 22.0	NS
Duration of pain	2.2 ± 1.0	2.2 ± 0.9	NS
Use of painkillers	1.91 ± 3.7	0.53 ± 1.1	0.044∗
Pain in VAS^a^	6.6 ± 0.4	6.3 ± 1.9	NS
MDQ scores^b^			
Pain	19.2 ± 6.0	18.9 ± 6.3	NS
Concentration	15.9 ± 8.2	14.8 ± 6.7	NS
Behavioral change	13.8 ± 6.2	11.9 ± 6.4	NS
Autonomic reaction	10.2 ± 4.2	9.4 ± 4.7	NS
Control	10.0 ± 3.9	9.3 ± 4.7	NS
Water retention	9.5 ± 4.0	9.3 ± 4.0	NS
Negative affect	18.9 ± 8.7	20.6 ± 9.5	NS
Arousal	6.0 ± 2.4	5.6 ± 1.1	NS
WHOQOL-BREF scores^c^			
Global	11.1 ± 2.2	11.8 ± 3.1	NS
Physiological domain	13.0 ± 2.0	13.3 ± 2.2	NS
Psychological domain	12.8 ± 2.1	13.1 ± 2.7	NS
Social domain	13.5 ± 1.6	14.1 ± 1.9	NS
Environmental domain	13.9 ± 1.6	14.3 ± 2.1	NS

^a^VAS refers to the Visual Analogue Scale; ^b^MDQ refers to the Menstrual Distress Questionnaire; ^c^WHOQOL-BREF refers to the World Health Organization Quality of Life-brief version. *P* values are calculated for the comparison of difference between endometriosis and primary dysmenorrhea groups by *χ* ^2^ or the independent *t*-test. ∗Significance at the *P* < 0.05 level.

**Table 3:** Means and standard deviation of major variable measured at baseline and weeks 4, 8, and 12.

Variable definition	Endometriosis				Primary dysmenorrhea				Time effect P value	Time ∗ group effect P value
	Baseline	Week 4	Week 8	Week 12	Baseline	Week 4	Week 8	Week 12		
Pain in VAS ^a^	6.6 ± 2.1	5.8 ± 2.5	5.2 ± 2.4	5.4 ± 2.3	6.3 ± 1.9	5.0 ± 2.8	4.6 ± 2.8	4.4 ± 2.7	0.000∗	0.698

Menstrual duration	2.9 ± 0.8	2.9 ± 0.8	2.9 ± 0.8	2.7 ± 0.8	2.7 ± 0.6	2.6 ± 0.7	2.6 ± 0.7	2.6 ± 0.7	0.250	0.779
Blood volume	36.4 ± 38.8	31.4 ± 32.4	30.7 ± 31.4	28.9 ± 31.2	26.1 ± 22.0	22.4 ± 16.6	18.0 ± 12.0	19.8 ± 14.8	0.001∗	0.760
Duration of pain	2.2 ± 1.0	2.3 ± 1.1	1.9 ± 1.0	2.1 ± 1.1	2.2 ± 0.9	2.0 ± 1.2	1.8 ± 1.1	1.8 ± 1.2	0.017∗	0.520
Use of painkillers	1.9 ± 3.7	1.7 ± 3.4	1.7 ± 3.3	1.9 ± 4.1	0.5 ± 1.1	0.5 ± 1.1	0.5 ± 0.9	0.4 ± 1.1	0.783	0.791

WHOQOL-BREF score^b^										
Global	11.1 ± 2.2	11.4 ± 2.3	11.5 ± 2.6	12.2 ± 2.0	11.8 ± 3.1	11.7 ± 3.0	11.7 ± 3.0	11.9 ± 3.1	0.046∗	0.208
Physiological domain	13.0 ± 2.0	12.6 ± 2.1	12.5 ± 2.3	12.8 ± 2.0	13.3 ± 2.2	13.5 ± 2.4	13.4 ± 2.1	13.7 ± 2.6	0.303	0.348
Psychological domain	12.8 ± 2.1	12.4 ± 2.1	12.2 ± 2.0	12.6 ± 2.0	13.1 ± 2.7	12.9 ± 2.3	12.7 ± 2.3	12.9 ± 2.4	0.013∗	0.707
Social domain	13.5 ± 1.6	13.0 ± 1.8	12.8 ± 1.7	13.0 ± 1.8	14.1 ± 1.9	13.7 ± 2.0	13.6 ± 2.1	13.7 ± 1.9	0.002∗	0.883
Environmental domain	13.9 ± 1.6	13.6 ± 1.8	13.3 ± 1.9	13.5 ± 1.9	14.3 ± 2.1	14.1 ± 2.2	13.6 ± 2.2	13.6 ± 2.2	0.000∗	0.698

^a^VAS refers to the Visual Analogue Scale; ^b^WHOQOL-BREF refers to the World Health Organization Quality of Life-brief version. Data presented with mean ± standard deviation; data adjusted for age. ∗Significance at the *P* < 0.05 level.

**Table 4:** Means and standard deviation of Menstrual Distress Questionnaire (MDQ) measured at baseline and weeks 4, 8, and 12.

Variable definition	Endometriosis				Primary dysmenorrhea				Time effect *P* value	Time ∗ group effect *P* value
	Baseline	Week 4	Week 8	Week 12	Baseline	Week 4	Week 8	Week 12		
Pain	19.2 ± 6.0	18.2 ± 6.2	18.2 ± 6.1	17.3 ± 6.0	18.9 ± 6.3	17.1 ± 7.6	15.3 ± 7.5	14.8 ± 7.6	0.000∗	0.046∗
Muscle stiffness	2.1 ± 1.4	2.2 ± 1.2	2.3 ± 1.4	2.1 ± 1.1	1.9 ± 1.1	2.1 ± 1.5	2.0 ± 1.3	1.9 ± 1.3	0.390	0.648
Headache	2.5 ± 1.7	2.6 ± 1.6	2.5 ± 1.6	2.5 ± 1.6	2.8 ± 1.8	2.9 ± 1.8	2.5 ± 1.7	2.3 ± 1.8	0.111	0.278
Cramps	4.4 ± 1.5	4.0 ± 1.6	3.6 ± 1.5	3.6 ± 1.6	4.4 ± 1.7	3.6 ± 1.6	3.2 ± 1.8	3.0 ± 1.7	0.000∗	0.233
Backache	3.7 ± 1.5	3.3 ± 1.5	3.5 ± 1.4	3.3 ± 1.4	3.3 ± 1.7	2.9 ± 1.7	2.6 ± 1.7	2.7 ± 1.6	0.004∗	0.227
Fatigue	3.8 ± 1.5	3.5 ± 1.5	3.7 ± 1.3	3.4 ± 1.0	3.9 ± 1.7	3.3 ± 1.8	2.9 ± 1.7	2.9 ± 1.5	0.000∗	0.037∗
General aches and pains	2.7 ± 1.5	2.6 ± 1.4	2.6 ± 1.4	2.4 ± 1.4	2.7 ± 1.5	2.2 ± 1.4	2.1 ± 1.4	2.1 ± 1.4	0.007∗	0.198
Concentration	15.9 ± 8.2	17.5 ± 8.1	16.6 ± 7.4	16.6 ± 7.1	14.8 ± 6.7	14.4 ± 7.1	13.5 ± 6.1	13.1 ± 6.1	0.377	0.221
Insomnia	2.4 ± 1.6	2.2 ± 1.5	2.1 ± 1.3	2.1 ± 1.2	2.0 ± 1.3	1.9 ± 1.1	2.0 ± 1.2	1.9 ± 1.2	0.660	0.834
Forgetfulness	1.7 ± 1.2	2.1 ± 1.3	1.9 ± 1.1	1.9 ± 1.1	1.9 ± 1.2	1.8 ± 1.2	1.7 ± 1.0	1.6 ± 1.0	0.348	0.126
Confusion	1.6 ± 1.0	2.0 ± 1.3	2.1 ± 1.1	2.1 ± 1.2	1.7 ± 1.0	1.8 ± 1.4	1.8 ± 1.2	1.7 ± 1.1	0.089	0.199
Lowered judgment	2.3 ± 1.4	2.5 ± 1.5	2.3 ± 1.3	2.1 ± 1.2	2.1 ± 1.3	1.8 ± 1.3	1.6 ± 1.0	1.6 ± 1.0	0.025∗	0.130
Difficulty concentrating	2.5 ± 1.6	2.6 ± 1.5	2.6 ± 1.6	2.6 ± 1.5	2.3 ± 1.5	2.4 ± 1.6	2.1 ± 1.4	2.1 ± 1.4	0.766	0.553
Distractible	2.0 ± 1.4	2.4 ± 1.5	2.1 ± 1.3	2.1 ± 1.2	1.9 ± 1.2	1.8 ± 1.1	1.6 ± 0.9	1.7 ± 0.9	0.356	0.368
Accidents	1.5 ± 0.9	1.6 ± 0.9	1.5 ± 0.9	1.6 ± 1.1	1.4 ± 0.8	1.3 ± 0.6	1.2 ± 0.5	1.3 ± 0.5	0.851	0.446
Lowered motor coordination	2.1 ± 1.7	2.1 ± 1.4	2.0 ± 1.3	1.9 ± 1.2	1.6 ± 1.1	1.6 ± 1.0	1.5 ± 1.1	1.3 ± 0.7	0.261	0.964
Behavioral change	13.8 ± 6.2	14.1 ± 5.8	13.5 ± 5.7	13.0 ± 5.2	11.9 ± 6.5	12.3 ± 6.6	10.8 ± 6.0	10.7 ± 5.8	0.037∗	0.778
Lowered school or work performance	2.8 ± 1.6	2.4 ± 1.2	2.4 ± 1.3	2.3 ± 1.1	2.0 ± 1.2	2.2 ± 1.4	2.1 ± 1.3	2.0 ± 1.2	0.186	0.142
Take naps	2.8 ± 1.7	3.1 ± 1.6	2.9 ± 1.5	2.7 ± 1.5	2.5 ± 1.6	2.6 ± 1.7	2.2 ± 1.4	2.4 ± 1.5	0.293	0.469
Stay at home	2.8 ± 1.6	3.2 ± 1.7	3.0 ± 1.5	3.0 ± 1.7	2.4 ± 1.7	2.6 ± 1.6	2.3 ± 1.3	2.2 ± 1.4	0.221	0.749
Avoid social activities	2.9 ± 1.7	2.9 ± 1.6	2.9 ± 1.6	2.8 ± 1.5	2.7 ± 1.9	2.5 ± 1.7	2.1 ± 1.5	2.2 ± 1.5	0.167	0.162
Decreased efficiency	2.6 ± 1.7	2.4 ± 1.5	2.3 ± 1.4	2.2 ± 1.1	2.2 ± 1.4	2.4 ± 1.5	2.1 ± 1.4	1.8 ± 1.2	0.026∗	0.501
Autonomic reaction	10.2 ± 4.2	8.8 ± 4.0	8.5 ± 3.7	8.5 ± 3.6	9.4 ± 4.7	7.3 ± 4.4	6.3 ± 3.0	6.1 ± 3.3	0.000∗	0.105
Dizziness, faintness	2.8 ± 1.7	2.4 ± 1.5	2.4 ± 1.3	2.6 ± 1.4	2.9 ± 1.7	2.7 ± 1.8	2.1 ± 1.4	2.0 ± 1.4	0.001∗	0.026∗
Cold sweats	3.0 ± 1.9	2.4 ± 1.6	2.2 ± 1.5	2.3 ± 1.4	2.6 ± 1.7	1.6 ± 1.4	1.5 ± 1.1	1.5 ± 1.1	0.000∗	0.661
Nausea, vomiting	2.7 ± 1.6	2.3 ± 1.4	2.0 ± 1.2	2.0 ± 1.0	2.3 ± 1.5	1.7 ± 1.1	1.4 ± 0.8	1.5 ± 1.0	0.000∗	0.876
Hot flashes	1.7 ± 1.1	1.7 ± 1.2	1.8 ± 1.1	1.7 ± 1.1	1.7 ± 1.5	1.4 ± 1.0	1.2 ± 0.6	1.1 ± 0.5	0.095	0.027∗
Control	10.0 ± 3.9	10.2 ± 4.1	10.4 ± 4.0	9.8 ± 4.0	9.3 ± 4.7	9.0 ± 4.7	7.9 ± 2.9	7.5 ± 2.7	0.005∗	0.014∗
Feeling of suffocation	1.6 ± 0.9	1.5 ± 0.7	1.5 ± 0.7	1.6 ± 0.8	1.4 ± 0.8	1.4 ± 0.8	1.4 ± 0.7	1.2 ± 0.5	0.907	0.249
Chest pains	2.1 ± 1.2	2.0 ± 1.2	2.1 ± 1.4	1.8 ± 1.2	1.9 ± 1.3	1.6 ± 1.1	1.4 ± 0.8	1.4 ± 0.8	0.008∗	0.243
Ringing in the ears	1.2 ± 0.5	1.3 ± 0.6	1.3 ± 0.6	1.3 ± 0.6	1.3 ± 0.8	1.2 ± 0.6	1.2 ± 0.6	1.1 ± 0.4	0.817	0.246
Heart pounding	1.8 ± 1.0	1.6 ± 0.9	1.6 ± 1.0	1.5 ± 0.7	1.7 ± 1.1	1.5 ± 1.0	1.1 ± 0.4	1.2 ± 0.5	0.000∗	0.057
Numbness, tingling	1.8 ± 1.3	1.7 ± 1.2	1.9 ± 1.4	1.8 ± 1.2	1.3 ± 1.0	1.2 ± 0.7	1.2 ± 0.4	1.1 ± 0.3	0.618	0.426
Blind spots, fuzzy vision	1.6 ± 1.0	2.1 ± 1.3	2.0 ± 1.3	1.8 ± 1.1	1.7 ± 1.5	2.0 ± 1.6	1.6 ± 1.3	1.5 ± 1.2	0.003∗	0.219
Water retention	9.5 ± 4.0	9.4 ± 3.7	9.4 ± 3.4	10.1 ± 4.0	9.3 ± 4.0	8.3 ± 4.3	7.9 ± 4.1	7.4 ± 3.3	0.157	0.009∗
Weight gain	2.0 ± 1.3	2.2 ± 1.4	2.0 ± 1.3	2.4 ± 1.4	1.7 ± 1.0	1.7 ± 1.0	1.8 ± 1.3	1.7 ± 0.8	0.487	0.144
Skin disorders	2.8 ± 1.6	2.7 ± 1.7	2.6 ± 1.4	2.8 ± 1.5	2.9 ± 1.6	2.8 ± 1.8	2.7 ± 1.8	2.6 ± 1.6	0.602	0.616
Painful breasts	2.6 ± 1.3	2.4 ± 1.2	2.6 ± 1.5	2.6 ± 1.3	2.8 ± 1.6	2.1 ± 1.5	1.9 ± 1.3	1.8 ± 1.3	0.007∗	0.007∗
Swelling	2.2 ± 1.3	2.1 ± 1.2	2.2 ± 1.4	2.3 ± 1.4	1.9 ± 1.4	1.7 ± 1.3	1.6 ± 1.1	1.4 ± 0.8	0.310	0.065
Negative affect	18.9 ± 8.7	18.5 ± 6.8	19.0 ± 7.7	17.7 ± 7.1	20.6 ± 9.5	17.2 ± 9.4	16.9 ± 9.0	15.8 ± 7.8	0.001∗	0.030∗
Crying	1.9 ± 1.4	1.8 ± 1.0	1.9 ± 1.0	2.0 ± 1.0	1.9 ± 1.1	2.0 ± 1.4	1.9 ± 1.4	1.9 ± 1.3	0.997	0.727
Loneliness	1.6 ± 0.9	1.7 ± 0.7	1.8 ± 1.0	1.7 ± 0.9	1.8 ± 1.2	1.5 ± 1.0	1.7 ± 1.0	1.5 ± 0.8	0.487	0.203
Anxiety	2.5 ± 1.5	2.2 ± 1.2	2.4 ± 1.3	2.2 ± 1.2	2.9 ± 1.8	2.1 ± 1.6	2.1 ± 1.4	2.1 ± 1.5	0.001∗	0.192
Restlessness	3.1 ± 1.7	2.8 ± 1.4	2.8 ± 1.5	2.6 ± 1.3	3.0 ± 1.8	2.3 ± 1.5	2.3 ± 1.5	2.1 ± 1.4	0.000∗	0.538
Irritability	3.0 ± 1.6	2.9 ± 1.5	2.9 ± 1.4	2.7 ± 1.5	3.4 ± 1.8	2.6 ± 1.7	2.5 ± 1.5	2.3 ± 1.4	0.001∗	0.047∗
Mood swings	2.9 ± 1.5	2.9 ± 1.4	2.9 ± 1.3	2.8 ± 1.2	3.4 ± 1.8	2.8 ± 1.7	2.6 ± 1.6	2.5 ± 1.5	0.004∗	0.060
Depression	1.9 ± 1.3	2.1 ± 1.2	2.1 ± 1.2	2.1 ± 1.1	2.3 ± 1.5	2.1 ± 1.4	1.9 ± 1.3	1.8 ± 1.2	0.325	0.053
Tension	2.0 ± 1.3	2.1 ± 1.1	2.2 ± 1.2	1.7 ± 1.0	2.0 ± 1.5	1.8 ± 1.4	1.8 ± 1.3	1.7 ± 1.2	0.071	0.301
Arousal	6.0 ± 2.4	6.4 ± 3.0	6.4 ± 2.4	6.4 ± 2.4	5.6 ± 1.1	5.7 ± 1.6	5.6 ± 1.8	5.4 ± 0.9	0.580	0.368
Affectionate	1.1 ± 0.3	1.4 ± 0.8	1.2 ± 0.5	1.2 ± 0.5	1.0 ± 0.2	1.1 ± 0.5	1.1 ± 0.7	1.0 ± 0.2	0.065	0.657
Orderliness	1.2 ± 0.5	1.3 ± 1.0	1.3 ± 0.6	1.3 ± 0.6	1.0 ± 0.2	1.2 ± 0.4	1.1 ± 0.7	1.1 ± 0.3	0.307	0.977
Excitement	1.2 ± 0.7	1.4 ± 0.7	1.5 ± 1.0	1.5 ± 0.9	1.5 ± 1.0	1.3 ± 0.8	1.2 ± 0.4	1.1 ± 0.3	0.745	0.000∗
Feeling of well-being	1.3 ± 0.8	1.1 ± 0.4	1.2 ± 0.5	1.3 ± 0.7	1.0 ± 0.0	1.0 ± 0.0	1.2 ± 0.6	1.1 ± 0.3	0.299	0.126
Bursts of energy, activity	1.2 ± 0.9	1.2 ± 0.6	1.3 ± 0.8	1.2 ± 0.7	1.0 ± 0	1.1 ± 0.3	1.1 ± 0.2	1.1 ± 0.4	0.817	0.817

Data presented with mean ± standard deviation. Data adjusted for age. ∗Significance at the *P* < 0.05 level.
